# Loss of TACC1 variant25 inducing cell proliferation and suppressing autophagy in head and neck squamous carcinoma

**DOI:** 10.1038/s41420-021-00777-6

**Published:** 2021-12-11

**Authors:** Pan Xu, Ran Zhao, Chen-Yang Zhang, Qian-Qian Zhang, Yong Wang, Jun Zhu, Wei-Wen Jiang

**Affiliations:** 1 Department of Oral Mucosal Diseases, Shanghai Ninth People’s Hospital, Shanghai Jiao Tong University School of Medicine, Shanghai, China; 2 College of Stomatology, Shanghai Jiao Tong University, Shanghai, China; 3 National Center for Stomatology, Shanghai, China; 4 National Clinical Research Center for Oral Diseases, Shanghai, China; 5 Shanghai Key Laboratory of Stomatology, Shanghai, China; 6 Department of Oral Surgery, Shanghai Ninth People’s Hospital, Shanghai Jiao Tong University School of Medicine, Shanghai, China; 7 CNRS-LIA Hematology and Cancer, Sino-French Research Center for Life Sciences and Genomics, State Key Laboratory of Medical Genomics, Rui Jin Hospital, School of Medicine, Shanghai Jiao Tong University, Shanghai, China; 8 Université de Paris 7/INSERM/CNRS UMR 944/7212, Equipe Labellisée No. 11 Ligue Nationale Contre le Cancer, Hôpital St. Louis, Paris, France

**Keywords:** Oral cancer, Macroautophagy, Cell growth

## Abstract

Transforming acidic coiled-coil containing protein1 (TACC1) is closely related to transcription, translation and centrosome dynamics. Dysregulation of TACC1 is associated with multiple malignancies. Alternative splicing (AS) of TACC1 produces multiple variants, which are of great significance in cancer biology. However, the expression and biological functions of TACC1 variants in head and neck squamous cell carcinoma (HNSCC) remain unclear. In this study, we found for the first time that TACC1 variants exhibited a characteristic expression pattern and that TACC1 variant25 (TACC1v25) was downregulated in HNSCC tissues and cell lines. Overexpression of TACC1v25 in Cal27 and Fadu cells significantly inhibited proliferation and promoted autophagy. Moreover, expression levels of nuclear pERK and p-mTOR were significantly decreased, while the expression of Beclin-1 and the LC3II/LC3I ratio were increased in TACC1v25-overexpressed Cal27 and Fadu cells. After the addition of AKT activator SC79 to TACC1v25-overexpressed Cal27 and Fadu cells, the autophagy levels were remarkably rescued. In conclusion, TACC1v25 inhibits HNSCC progression through the ERK and AKT/mTOR pathways by inhibiting proliferation and increasing autophagy. TACC1v25 might have potential use as a tumour suppressor in HNSCC.

## Introduction

Head and neck squamous cell carcinoma (HNSCC) is one of the most common malignant tumours worldwide, accounting for 2.5% of all new cancer cases and 1.9% of all cancer deaths annually [1]. Although great progress has been made in surgical techniques, radiation and chemoradiation treatment, the overall 5-year survival rate of HNSCC patients remains only 50–60%. Therefore, further study of the underlying molecular biological mechanisms of HNSCC development is necessary for more effective treatment.

Alternative splicing (AS) is frequently observed in human multiexon genes [2, 3]. It is closely linked to human disease [4] and is a hallmark of cancer [3]. AS events include seven splicing patterns: alternate acceptor site (AA), alternate donor site (AD), alternate promoter (AP), alternate terminator (AT), exon skipping (ES), mutually exclusive exons (ME) and retained intron (RI) [5]. Differences in splicing patterns lead to different gene expression patterns and phenotypes between individuals [6]. AS profile data have demonstrated that AS is closely linked to HNSCC [6].

Transforming acidic coiled-coil containing protein1 (TACC1), a member of the TACC family, has a feature of the TACC gene family, namely, a carboxy-terminal large coiled-coil domain (TACC domain). TACC1 is closely related to the processes of transcription, translation and centrosome dynamics by interacting with a variety of complex components [7, 8]. Recent studies have shown that dysregulation of TACC1 seems to be associated with the occurrence of multiple malignancies, such as rhabdomyosarcoma, breast cancer, gastric cancer, leukaemia and ovarian cancer [3, 9–13]. Based on variable transcription start sites and alternative exon usage, a previous study described TACC1A-F [2] and later TACC1G-I, S [14], TACC1-J [15] and TACC1-K [8]. TACC1-A*, -J* and -S* were predicted but have not yet been identified [15]. In addition, TACC1 has been classified into long and short TACC1 isoforms according to whether two serine- and proline-rich AZU-1(SPAZ) motifs are contained in exons 2 and 3, respectively [16, 17]. As of December 2019, in GenBank, a total of 44 mRNA transcript variants have been described in human TACC1 (Gene ID:6867), including 31 encoding transcript variants, seven noncoding transcript variants and six predicted transcript variants.

TACC1 and its variants are suggested to exhibit temporal and spatial expression patterns in human organs [15]. Changes in TACC1 mRNA splicing patterns in cancer cells may interfere with TACC1 function [2]. In mouse breast tumours, as an important potential activating factor in tumour formation, full-length TACC1 expression results in increased phosphorylation of ERK and PKB. Full-length TACC1 positively regulates the Ras and mTOR pathways, and cooperates with them in colony formation, cell survival and tumorigenesis [18]. On the other hand, the AKT/mTOR pathway is a critical regulator of autophagy, which can determine the survival and death of cells and plays an important role in tumorigenesis [19].

In our current study, we first found that a characteristic expression pattern of TACC1 variants and TACC1 variant25 (TACC1v25) was downregulated in HNSCC tissues and cell lines. Moreover, overexpression of TACC1 isoform4, which is encoded by TACC1v25, inhibited proliferation and increased autophagy in HNSCC lines via the ERK and AKT/mTOR signalling pathways in HNSCC.

## Results

### TACC1 variants exhibiting differential expression between HNSCC and NHOK

**Fig. 1:**
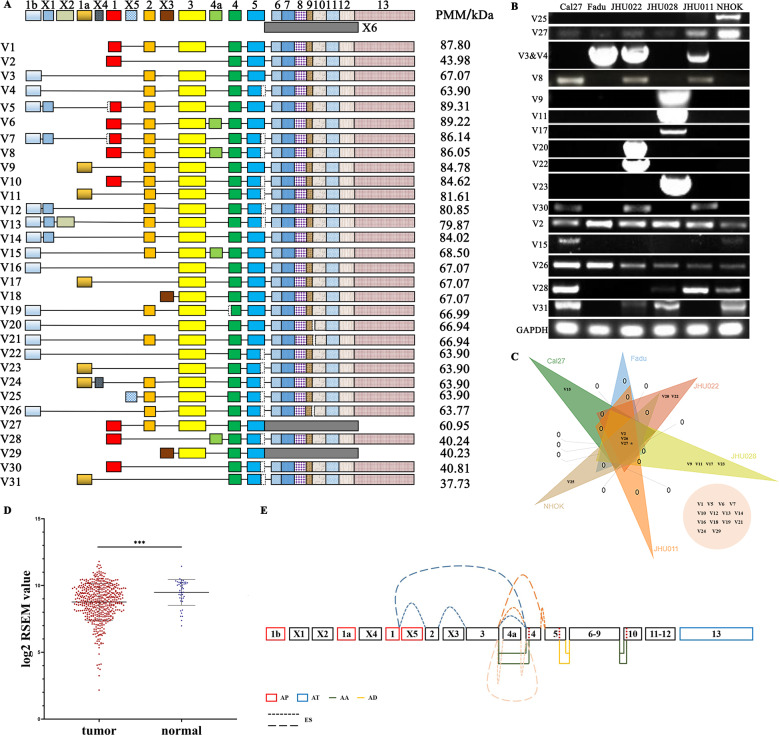
Expression of TACC1 variants in HNSCC. **A** Diagrammatic representation of TACC1 variants. In GenBank, human TACC1 (Gene ID:6867) mRNA has a total of 31 encoding transcript variants. **B** Expression analysis of TACC1 variants in NHOK and HNSCC cell lines by RT-PCR. TACC1 v25 and 27 exhibit higher expression in NHOK cells. **C** Venn diagram of describing the differential expression patterns of TACC1 variants in HNSCC cell lines and NHOK cells. *TACC1v27 was expressed in all cell lines, but there was a significant difference in expression levels between HNSCC lines and NHOK cells. Round circle, negative variants in all cells. **D** Analysis of TACC1v25 expression in HNSCC samples by TCGA. In normal tissue samples, TACC1v25 showed higher expression than that in primary HNSCC tissue samples. **E** Analysis of AS events associated with TACC1 in HNSCC by TCGA. Box with colour, exon; blank box, lacking region of exon; arrow, location of primers. T, HNSCC samples; N, normal controls (^***^P < 0.001). Note: The nomination of TACC1 exons was in line with ref. [15]. Exons X1-X6 were named by our study.

According to GenBank, the human TACC1 gene has a total of 31 transcript variants that encode protein (Fig. 1A). Because TACC1 variants have been suggested to exhibit temporal and spatial expression patterns in human organs [15], we performed RT-PCR to compare the characteristics of their expression in HNSCC cell lines and normal human oral keratinocytes (NHOKs). Higher expression in NHOKs was detected in variants 25 and 27. Ten TACC1 variants, including variants 3, 4, 8, 9, 11, 17, 20, 22, 23 and 30, were only expressed in HNSCC cell lines. Variants 2, 15, 26, 27, 28 and 31 were expressed in both NHOK and HNSCC cell lines (Fig. 1B). Variants 2, 26 and 27 were expressed in each cultured cells, however, variant 27 was extremely higher expressed in NHOKs than that of in HNSCC cell lines (Fig. 1C). Furthermore, TACC1v25 was significantly highly expressed in normal tissue compared to primary HNSCC by using the online tool TSVdb (Fig. 1D), which was consistent with our findings. Meanwhile, using the TCGA database, we extracted AS events for TACC1 in HNSCC patients. Using SpliceSeq, 11 AS events in TACC1 were identified in HNSCC, which including AP in exons 1, 1a, X5 and 1b, AT in exon 13, AA in exons 4 and 10, AD in exon 5, and ES in exons 2, 3, 4a and 4, respectively (Fig. 1E and Supplementary Table S3).

### Expression of endogenous TACC1 protein in HNSCC and NHOK

**Fig. 2:**
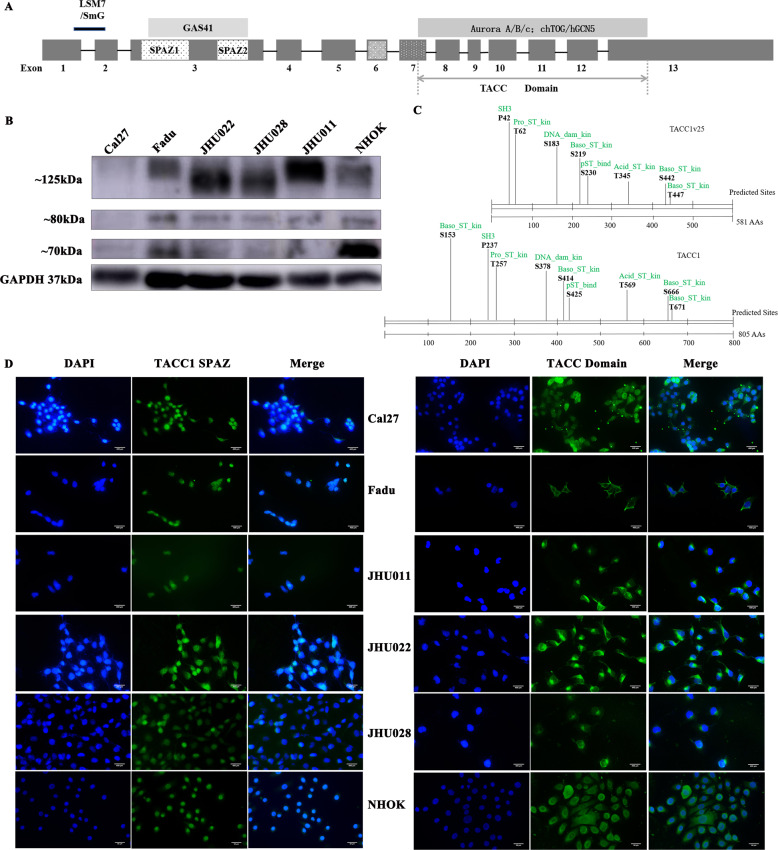
Expression of endogenous TACC1 protein in HNSCC. **A** Schematic representation of TACC1 domains. This schematic diagram shows published TACC domain. **B** Western blot analysis. Because the anti-TACC1 domain antibody identifies all TACC1 variants proteins, stronger bands at ~70 and ~80 kDa in NHOK were TACC1v25 and/or 27 combined with RT-PCR. **C** Scansite analysis. Phosphorylation sites were predicted in TACC1v25 and full-length TACC1. The black words represent the possible phosphorylation site, while the green words represent the kinase or possible binding structure domain. **D** Immunofluorescence analysis. TACC1 variants include SPAZ domain encoding proteins that are primarily expressed in nucleus and perinucleus; however, TACC1 variants encoding protein containing TACC domains express in the nucleus, perinuclear region and cytoplasm. There was no significant difference of expression among the lines.

To determine the expression of endogenous TACC1 protein in HNSCC and NHOK, we performed western blotting using an anti-TACC1 antibody (anti-TACC domain). The predicted molecular weight of TACC1v27 is 60.95 kDa, and that of TACC1v25 is 63.90 kDa. The apparent molecular mass of the endogenous TACC1 protein was much higher than the predicted one, which could be caused by conformational structures of the SPAZ and coiled-coil motifs and/or the post-translationally modified form (Fig. 2A, B), possibly a phosphorylated form of TACC1 [16]. Post-translational modification of proteins plays an important role in cellular regulation and phosphorylation is one of the most important type of post-translational modification [20]. We used Scansite to analyse phosphorylation sites of TACC1v25 and full-length TACC1 encoding protein. There were 8 (P42, T62, S183, S219, S230, T345, S442, T447) and 9 sites (S153, P237, T257, S378, S414, S425, T569, S666, T671) in TACC1v25 and full-length TACC1 protein, respectively (Fig. 2C). Combined with the RT-PCR results, stronger bands were detected at ~70 and ~80 kDa in NHOK, most likely it was TACC1v25 (encoded by isoform 4) and/or 27 (Fig. 2B). The raw Fig. 2B is displayed in Supplementary Fig. S1.

To locate the expression of the TACC1 variants, immunofluorescence assay (IF) was used to detect the expression of TACC1 in HNSCC cell lines and NHOK cells using two kinds of TACC1 antibodies including antibodies which are specific to the TACC1 SPAZ domain and TACC domain. The results revealed that TACC1 was expressed in the cell nucleus, perinucleus and cytoplasm in both HNSCC cell lines and NHOK cells using the TACC1 domain antibody (Fig. 2D). We also observed that TACC1, including the SPAZ domain, was primarily expressed in the nucleus and perinucleus (Fig. 2D).

### Ectopic expression of TACC1v25 inhibiting cell proliferation in HNSCC

**Fig. 3:**
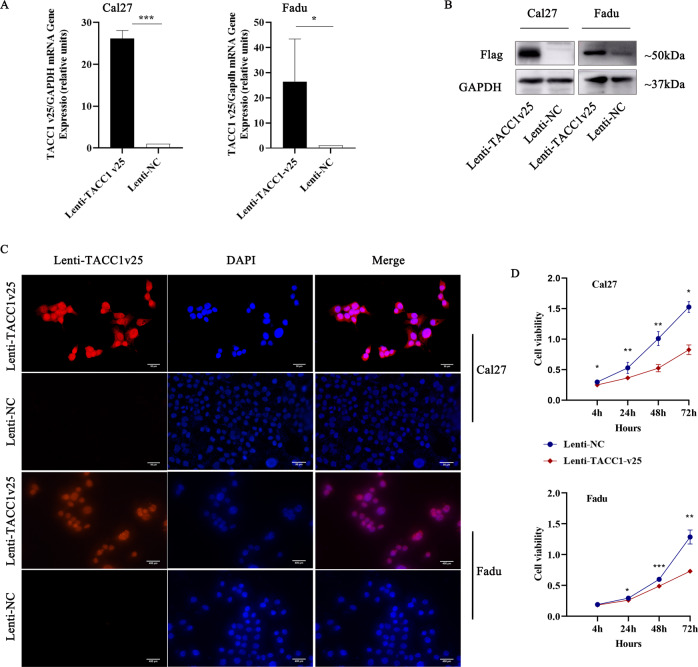
TACC1v25 inhibits cell proliferation in HNSCC. RT-qPCR (**A**) and western blot (**B**) verifying established Lenti-TACC1v25-Cal27/-Fadu cells. **C** Subcellular localization of ectopic TACC1v25 in Lenti-TACC1v25-Cal27/-Fadu cells (original magnification 400×). TACC1v25 (anti-Flag) was observed not only in the nucleus, but also in perinucleus membranes and cytoplasm by IF. **D** Effect of TACC1v25 on proliferation as determined by CCK8 assay. Cell proliferation was inhibited in Lenti-TACC1v25-Cal27/Fadu cells compared to NC group. ^*^P < 0.05, ^**^P < 0.01, ^***^P < 0.001.

According to PubMed, TACC1 variants 4, 22, 23, 24 and 25 encode an identical protein isoform 4. Considering that variants 4, 22 and 23 are expressed in the JHU011, JHU022 and JHU028 cell lines, we chose Cal27 and Fadu lines as cell models to eliminate the false-positive effects of these variants. We generated Cal27 cells and Fadu cells that stably overexpressed either Lenti-TACC1v25 or Lenti-NC (Fig. 3A, B) and then an anti-Flag antibody was used to determine the subcellular location of overexpressed TACC1v25 in Cal27 and Fadu cells (Fig. 3C). Strong levels of TACC1v25 were observed in nucleus, perinucleus membranes and cytoplasm by IF (Fig. 3C). To determine whether TACC1v25 acts as a tumour suppressor in HNSCC, proliferative activity was assessed in stable Cal27 cells and Fadu cells, and found that TACC1v25 decreased cell proliferation in both Lenti-TACC1v25-Cal27 and in Lenti-TACC1v25-Fadu cells (Fig. 3D).

### Ectopic expression of TACC1v25 affecting the phosphorylation levels of ERK in HNSCC

**Fig. 4:**
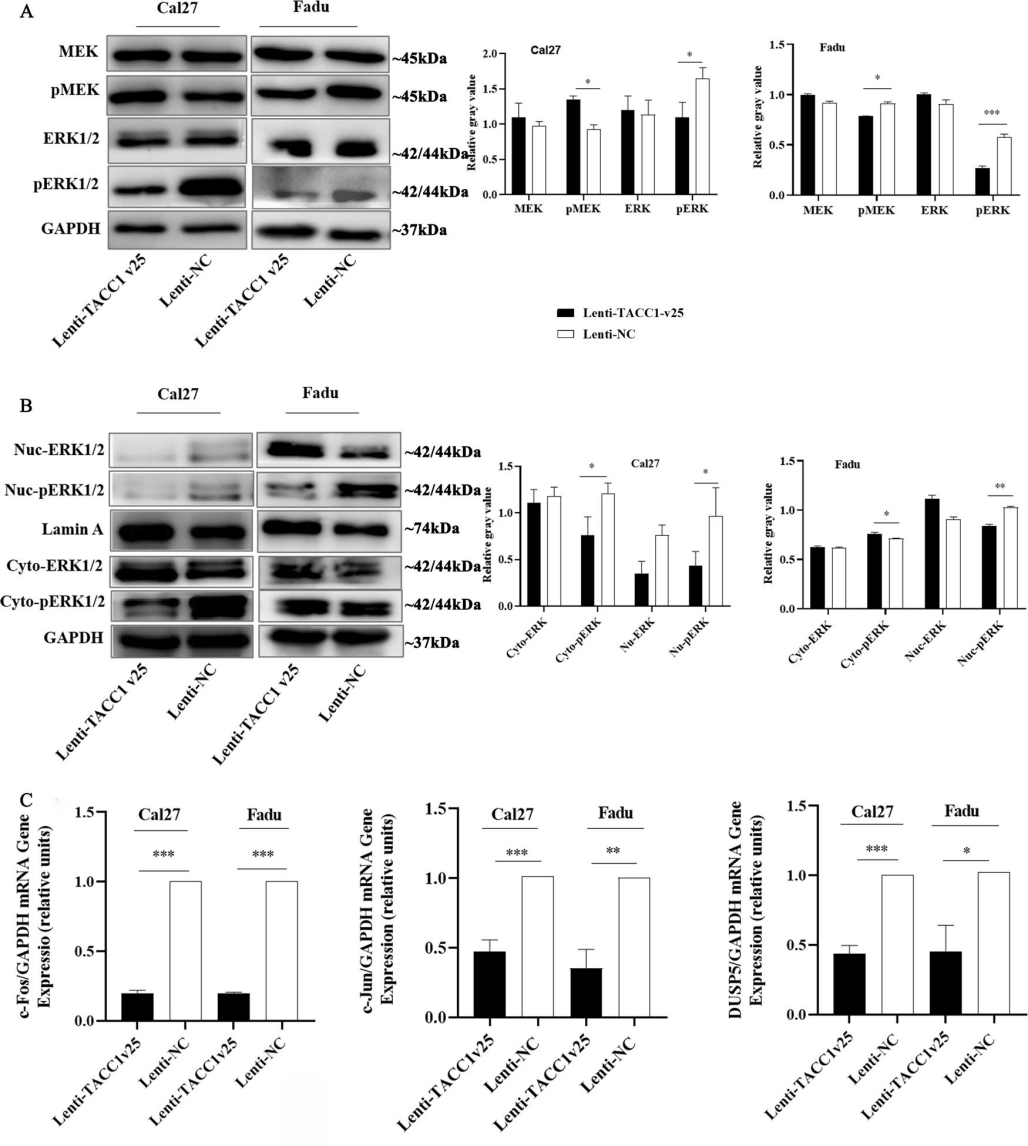
TACC1v25 reduces the phosphorylation of ERK in HNSCC. **A** Western blot analysis of total phosphorylation levels of the ERK pathway. Total expression of ERK was unchanged but expression of pERK was decreased in established Lenti-TACC1v25-Cal27/-Fadu cells. **B** Western blot analysis of nucleus and cytoplasmic phosphorylation level of ERK. The expression of pERK in nucleus is decreased in Lenti-TACC1v25-Cal27/-Fadu cells. The expression of cytoplasmic pERK is greatly decreased in Lenti-TACC1v25-Cal27 cells; however, it is slightly higher in Lenti-TACC1v25-Fadu cells. **C** Expression of downstream genes of the ERK pathway, c-Fos and c-Jun, are significantly downregulated in Lenti-TACC1v25-Cal27/-Fadu cells, and the ERK-specific inducible ribozyme, DUSP5, is also decreased. Cyto., Cytoplasm; Nuc., Nucleus. ^*^P < 0.05, ^**^P < 0.01, ^***^P < 0.001.

It was previously reported that activated ERK promotes cell proliferation by phosphorylating multiple substrates [19]. According to our results, total expression of ERK was unchanged but expression of ERK phosphorylation in Cal27 and Fadu cells stably expressing TACC1v25 was decreased (Fig. 4A). Activated ERK performs different functions in the cytoplasm and nucleus, therefore, we extracted nuclear and cytoplasmic proteins. Both in the cytoplasm and in the nucleus, pERK was expressed at lower levels in Lenti-TACC1v25-Cal27 cells. However, in Lenti-TACC1v25-Fadu cells, the pERK levels in the cytoplasm were increased, but they were decreased in the nucleus (Fig. 4B). In contrast, pMEK levels were increased and decreased in Lenti-TACC1v25-Cal27 and Lenti-TACC1v25-Fadu cells, respectively. We further examined the downstream targets of ERK. The expression of both c-Fos and c-Jun was significantly downregulated in Lenti-TACC1v25-Cal27/Fadu cells (Fig. 4C). DUSP5, an ERK-specific inducible ribozyme, modulates the duration and intensity of ERK phosphate activation in the MAPK cascade [21]. We found that DUSP5 mRNA was significantly downregulated in stably expressing TACC1v25-Cal27/Fadu cells (Fig. 4D).

### Ectopic expression of TACC1v25 leading to increased autophagy but no activity of apoptosis in HNSCC

**Fig. 5:**
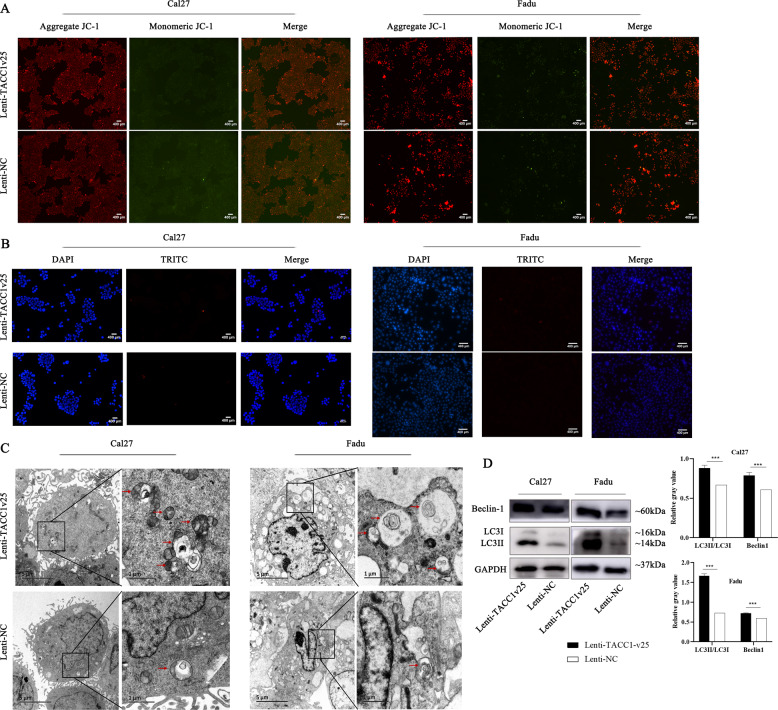
TACC1v25 increases autophagy but no changes apoptosis in HNSCC cells. **A** Apoptosis detection using JC-1 assay. There was no significant activity of early apoptosis in Lenti-TACC1v25-Cal27/-Fadu cells and their controls. (original magnification 100×). **B** Apoptosis detection by TUNEL assay. Late apoptosis was not observed in Lenti-TACC1v25-Cal27/-Fadu cells and their controls. (original magnification 200×). **C** Autophagosome detection by TEM. The number of autophagosomes is increased in Lenti-TACC1v25-Cal27/-Fadu cells. **D** Detection of autophagy-related proteins by western blot. Autophagic markers, LC3II/I and Beclin-1 are increased in stably expressing TACC1v25 cells. Blue, DAPI; red, Apoptosis cells. ^*^P < 0.05, ^**^P < 0.01, ^***^P < 0.001.

Apoptosis is classified as type I programmed cell death, and decreased activity of apoptosis is a hallmarker of cancer. However, we observed no significant activity of early/late apoptosis (Fig. 5A, B) in stably expressed TACC1v25-Cal27/Fadu cells. It has been reported that in non-apoptotic cells, autophagy might induce non-apoptotic programmed cell death [22]. To determine whether TACC1v25 affects autophagy, we performed transmission electron microscopy (TEM) analysis. Increased autophagosomes were observed in stably expressed TACC1v25-Cal27/Fadu cells than that in control cells (Fig. 5C). Additionally, the autophagic markers, LC3II/I and Beclin-1 were increased in stably expressed TACC1v25-Cal27/Fadu cells (Fig. 5D).

### Ectopic expression of TACC1v25 inhibiting the phosphorylation levels of AKT/mTOR in HNSCC

**Fig. 6:**
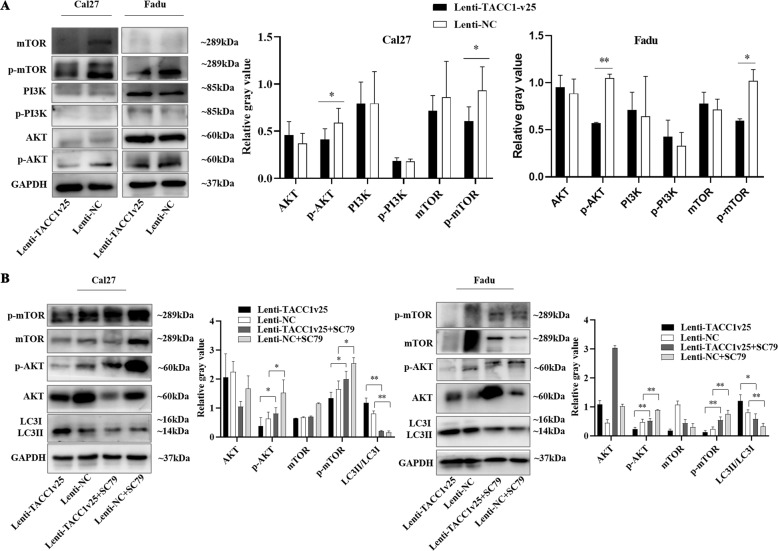
TACC1v25 inhibits activation of the PI3K/AKT/mTOR signalling pathway. **A** Western blot analysis of PI3K/AKT/mTOR. Expression of p-AKT and p-mTOR is decreased in Lenti-TACC1v25-Cal27/-Fadu cells. **B** After addition of the AKT activator, SC79, expression levels of p-AKT and p-mTOR are increased, and LC3II/LC3I is decreased in Lenti-TACC1v25-Cal27/-Fadu cells. ^*^P < 0.05, ^**^P < 0.01, ^***^P < 0.001.

There is evidence suggesting that full-length TACC1 activates the phosphorylation of AKT [23, 24], but it is not clear whether TACC1v25, a short-form TACC1 variant, regulates the AKT pathway. Therefore, expression of p-AKT, p-PI3K and p-mTOR, key proteins in the AKT/mTOR pathway, was examined. Expression of p-AKT and p-mTOR was significantly decreased in stably overexpressing TACC1v25-Cal27/Fadu cell lines (*P* < 0.05), but there was no change in the total AKT, PI3K, mTOR or p-PI3K expression (Fig. 6A). To verify whether TACC1v25 regulates autophagy through the AKT/mTOR pathway, autophagy was measured after the addition of SC79 (an AKT activator; Beyotime, Shanghai, China). We noticed that the LC3II/LC3I ratio and p-AKT/p-mTOR were reduced and increased, respectively, in SC79-treated Lenti-TACC1v25-Cal27/Fadu cells (Fig. 6B).

### Ectopic expression of TACC1v25 suppressing tumour growth in vivo

**Fig. 7:**
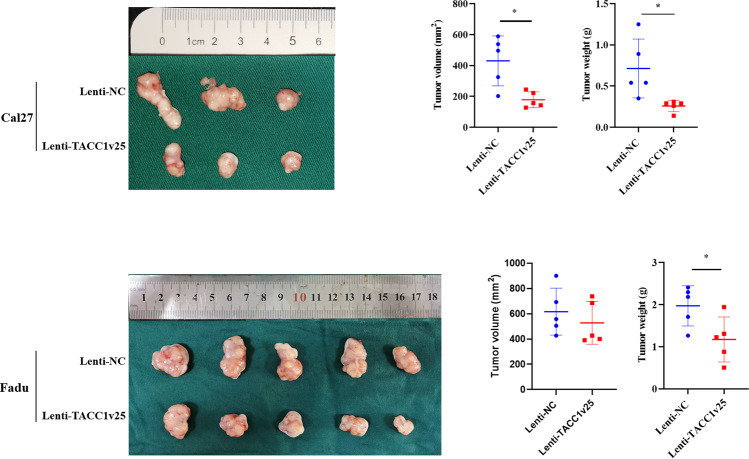
TACC1v25 inhibits the tumour growth in vivo. The weight and volume of tumours in the Lenti-TACC1v25-Cal27 group are significantly less than those in the Lenti-NC-Cal27 group (*N* = 5). In Lenti-TACC1v25-Fadu group, weight is significantly decreased, but there is no significant change in tumour volume. ^*^P < 0.05.

An in vivo tumorigenicity assay in nude mice revealed that the weight and volume of tumours in the Lenti-TACC1v25-Cal27 group and the weight of tumours in the Lenti-TACC1v25-Fadu group were significantly less than those in the Lenti-NC group. (Fig. 7).

## Discussion

In humans, TACC1 is suggested to have temporal and spatial expression patterns with specific combinations of variants [5, 15, 25]. AS events are related to carcinogenesis; moreover, the importance of AS events in HNSCC has been emphasized at the genomic or transcriptional level [15, 26]. In this study, we focused on differential TACC1 variants in HNSCC. We demonstrated that differentially expressed TACC1v25 exerted antiproliferation effects by altering the phosphorylation of ERK and regulated autophagy via the AKT/mTOR pathway in HNSCC.

The naming and classification of TACC1 variants have been changed over time [8, 14, 15]. Previous TACC1A, E, H, I, J, K and S are currently known as TACC1 variants 1, 3, 16, 9, 30, 10 and 2, respectively, based on the structure of the mRNA sequences of TACC1 variants. Known as the primary mode of genetic regulation in eukaryotes, the diversity of splicing patterns is responsible for the diversity of genomic proteins [27]. Protein isoforms produced by AS play an important role in cancer progression including cell proliferation, cell invasion and methylation defects [6]. The genome-wide analysis of AS in HNSCC using the TCGA program revealed a landscape of AS events related to carcinogenesis and its clinical significance [5]. In the current study, we extracted HNSCC-RNA-seq data from TCGA database and focused on AS in TACC1. Additionally, expression of TACC1v25 was validated using TSVdb-RNA-seq from patients with HNSCC. Among the 11 AS events identified, we noticed that the AP event was most likely associated with exon X5, which was only found in TACC1v25. This observation is consistent with previous studies in which differential promoter usage with AS was applied to the TACC1 gene [5].

The functions of different TACC1 variants are not entirely clear. Compared to full-length TACC1, the region deleted in TACC1v25 is exon 1 including the binding site for LSm7/SmG, which is related to RNA processing. Interestingly, the TACC1 isoforms expressed in human breast cancer cells do not interact with the histone acetyltransferase pCAF [28], although all human TACC proteins can directly interact with this histone acetyltransferase in vitro. Conte et al. suggested that this might be due to the proposed function of exon 1 via its interaction with LSm7 and SmG28. Combining TACC1v25 uniquely expressed in the HOK line, we assumed that in HNSCC, TACC1v25 possibly acts as a tumour suppressor. Although TACC1v25 did not exert its function in apoptosis, it decreased cell proliferation and regulated autophagy in HNSCC.

Except for the helical structure of the TACC domain at the carboxy terminus of TACC1, serine and proline are densely distributed in other regions, forming many SP repeats. The hydroxyl group of serine and hydroxylated proline can dehydrate with the phosphate group to form phosphate ester, which is known as phosphorylation [29]. Therefore, these dense SP repeats in TACC1 provide favourable conditions for phosphorylation. Post-translational modification of proteins plays an important role in cell process [20]. Phosphorylation modification is most widely investigated. Both Conte and Gabillard found that TACC1 is phosphorylated [6, 16, 30]. Gabillard et al. revealed that TACC1 can be phosphorylated as a substrate for Aurora C at tryptophan, at site 228 [30]. In our data, the apparent molecular mass of the TACC1 protein by western blot was much higher than the predicted one, which could result from the structures of the SPAZ and coiled-coil motifs and/or post-translationally modified forms, likely a phosphorylated form of TACC1 [16].

The RAS/RAF/MEK/ERK signalling cascade is critical for intercellular and intracellular communication, regulating cell functions, such as cell proliferation, growth, survival and differentiation [31], in which cell proliferation is primarily mediated by ERK activity in the nucleus [32]. As a downstream transcription factor of ERK, c-Fos plays an important role in tumorigenesis and is an important inducer of proliferation [32]. Blocking the ERK cascade is considered to be the primary goal in the treatment of many cancers resistant to RAF and MEK inhibitors. Studies have shown that ERK directly phosphorylates hundreds of substrates located in the cytoplasm, various organelles or nucleus [33]. ERK-mediated phosphorylation of the substrate alters the results of the signal [34]. Lauffart believed that TACC1 binds to FHL protein family members, regulates the phosphorylation of ERK and affects the intracellular localization of phosphorylated ERK, affecting cell differentiation and proliferation [7]. This also suggests that TACC1 affects cellular function through the Ras/Raf/MEK/ERK pathway. Our results further demonstrated that TACC1v25 plays a role in decreasing the phosphorylation of ERK, downregulating its downstream transcription factor c-Fos, and ultimately inhibiting cell proliferation.

**Fig. 8:**
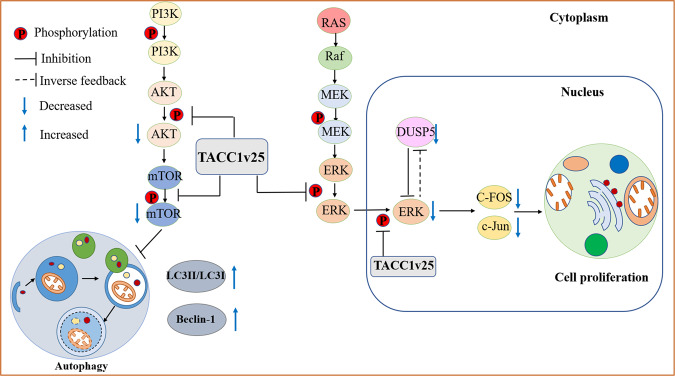
Schematic of the role of TACC1v25 in HNSCC. Ectopic TACC1v25 inhibits the expression of overall pERK and nucleus pERK. It decreases the expression of downstream genes, such as c-Fos and c-Jun, resulting in cell proliferation inhibition. Meanwhile, the ERK-specific inducible ribozyme, DUSP5, is also downregulated. On the other hand, ectopic TACC1v25 downregulates p-AKT and p-mTOR resulting in an increase in the autophagosome and autophagic protein markers LC3II and Beclin-1 in HNSCC cell lines.

The activation of ERK substrate molecules generates a feedback loop that controls the ERK signalling pathway in many ways and plays a vital role in maintaining cellular homeostasis under physiological conditions [34]. In contrast, activated ERK1/2 inhibits the phosphorylation of its upstream factors and kinases, such as MEK and Raf, blocking signalling of the pathway. In addition, activated ERK1/2 also stimulates DUSP dephosphorylation to regulate this pathway [35]. Based on our results, TACC1v25 inhibits phosphorylation of ERK and reduces expression of pERK, which has the following two effects: (1) decreased cytoplasmic pERK stimulates the phosphorylation of its upstream factors and kinases, thereby phosphorylating MEK (in Lenti-TACC1v25-Cal27; Fig. 8); and (2) decreased pERK reduces the expression of downstream targets c-Fos and c-Jun. While affecting cell proliferation, it also negatively regulates DUSP, thereby weakening the dephosphorylation of ERK by DUSP5 (Fig. 8). Through regulation of this negative feedback loop, ERK phosphorylation reaches a dynamic balance [35]. However, the pMEK levels of Lenti-TACC1v25 Cal27 and Fadu cells were opposite, which may be caused by the different mutations of p53 in two cell lines. Studies have showed that p53 mutation (codon 248 and 273 mutations) exist in Fadu cells, and p53 expression is only 50% of that in normal mucosa [36]; while Cal27 cells have a p53 mutation in codon 193 (A → T) [37]. Recent studies have shown that p53 can be an upstream activator to regulate MAPK signalling [38] and inactivation of ERK1/2 by p53 leads to the induction of apoptosis [38]. A feedback role exists between ERK and MEK in the RAS/RAF/MEK/ERK pathway [35], which may explain the diversity in pMEK expression between Cal27 and Fadu cell lines stably expressing TACC1v25. Further study is needed on the relationship between TACC1v25 and p53.

Autophagy plays an important role in cancer [39]. A recent study suggested that impaired autophagy, and the expression of LC3B are correlated with reduced overall and disease-specific survival in oral squamous cell cancer [40]. Knockdown of Beclin-1, a marker of autophagy, promotes the proliferation, migration and invasion of primary oral cancer cell lines. Inhibition of autophagy may result in a more aggressive cancer phenotype [41]. In our study, autophagy and Beclin-1 expression were both increased in TACC1v25 overexpressing HNSCC lines with deceased cell proliferation, suggesting that TACC1v25 may be useful against a more aggressive cancer phenotype. On the other hand, autophagy can both inhibit and promote apoptosis to affect the occurrence and development of cancers [39]. Yu et al. demonstrated that autophagic cell death was induced when apoptosis was inhibited [42]. Han et al. thought that an autophagic response was developed in some apoptosis-defective tumour cells due to various forms of stress [43]. In the current study, autophagy was increased but apoptosis was unchanged in response to TACC1v25 overexpression. It is not clear whether there are some kinds of stress in Cal27 and Fadu cells, and/or whether overexpressing TACC1v25 alone is unable to alter the activity of apoptosis in these two cell lines.

The PI3K/Akt/mTOR signalling pathway is an important pathway that regulates cell survival, under physiological and pathological conditions [44, 45]. Evidence has suggested that components of the AKT/mTOR pathway are intrinsic factors for carcinogenesis [19]. Alterations in the AKT/mTOR pathway were confirmed in TCGA HNSCC samples [46]. Tobacco, an HNSCC risk factor, has been reported to induce activation of the AKT/mTOR pathway [47]. mTOR, one of the major downstream targets of AKT, is also the major negative regulator of autophagy. It was reported that in mouse cancer, full-length TACC1 promotes the phosphorylation of AKT [18]. It is not surprising that TACC1v25, a short variant of TACC1, interacts with the AKT/mTOR pathway. However, in contrast to full-length TACC1, p-AKT/p-mTOR expression was decreased in Cal27/Fadu cells stably overexpressing TACC1v25. After treatment with an AKT activator in Lenti-TACC1v25 cells, the decreased p-mTOR and increased autophagy were recused. Taken together, these results indicate that TACC1v25 induces autophagy by inhibiting the AKT/mTOR pathway (Fig. 8).

In summary, AS variants of TACC1 are differentially expressed between NHOKs and HNSCC cell lines. Loss of TACC1v25 results in cell proliferation by increasing ERK phosphorylation in HNSCC. The characteristic expression patterns of TACC1 variants suggest that TACC1 is linked to HNSCC and might play an important role in oral carcinogenesis.

## Materials and methods

### Cell culture

The JHU-011, JHU-022, JHU-028 and Fadu human HNSCC lines were gifts from Dr. Califano at the Johns Hopkins University and were cultured in RPMI 1640 (Thermo Scientific, USA) supplemented with 10% FBS, 2 mmol/l L-glutamine, 100 U/ml penicillin and 100 g/ml streptomycin at 37 °C in a humidified 5% CO_2_ atmosphere. The Cal27 cell line was obtained from American Type Culture Collection (ATCC, Rockville, MA, USA) and was cultured in DMEM (Thermo Scientific) with 10% fetal calf serum at 37 °C and 5% CO_2_.

NHOKs were derived from the normal oral mucosa of a patient undergoing an impacted tooth extraction at the Department of Oral Surgery, Shanghai 9th People’s Hospital and were cultured with KSF medium (ScienCell, CA, USA) and penicillin/streptomycin solution. This study was approved by the institutional review board and signed informed consent was obtained from all participants (#SH9H-2019-T112-1).

### Plasmids and cell transfection

The TACC1v25 coding sequence (NM_001352798.1) was inserted into the pLenO-GTP-C-3XFlag/PGMLV-CMV-MCS1-3xFlag-PGK-Puro (with green fluorescence) and PGMLV-CMV-MCS1-PGK-Puro/PGMLV-CMV-MCS1-3xFlag-PGK-Puro-TACC1v25 plasmids to generate TACC1v25-negative and TACC1v25-expression vectors, respectively (Invitrogen Life Technologies, Carlsbad, CA, USA). Cal27 and Fadu cells were infected with lentiviral vectors. After undergoing 5 μg/ml puromycin selection, stable Lenti-TACC1v25-Cal27/Fadu and Lenti-NC-Cal27/Fadu cells were generated.

### RNA extraction, RT-PCR and RT-qPCR

Total RNA was extracted using TRIzol Reagents (Life Technologies, USA) according to the manufacture’s instructions and was reverse transcribed into cDNA using the PrimeScript™ 1st Strand cDNA Synthesis Kit (Takara, Japan). The Premix TaqTM (Takara, Japan) was used for PCR to detect specific variants in a reaction with 30 cycles of 98 °C for 10 s/kb, 55 °C for 30 s/kb and 72 °C for 1 min/kb. Sequence analysis of RT-PCR agarose gel products was performed to verify the TACC1 variants (Sangon Biotech, China).

Two-step RT-qPCR was performed using the PrimeScript™ RT Reagent Kit (Takara, Japan) and TB Green Premix Ex Taq (Takara, Japan) according to the manufacturer’s instructions. All samples were run in triplicate. GAPDH was used as an internal control and relative expression levels were determined using the comparative CT method (2^−∆∆CT^). All primers’ information is shown in the Supplementary Table S1.

### Western blotting

Cells were collected and lysed in ice-cold RIPA lysis buffer (Beyotime, Shanghai, China). The supernatant was collected after centrifugation at 14,000*g* for 5 min. Then, 20 µg of total protein was separated on 6–15% SDS-PAGE gels, and the proteins in the gel was transferred to a 0.22 μm PVDF membranes (Millipore, USA). Membranes were incubated with primary antibodies against TACC1, LC3B, Beclin-1, AKT, p-AKT, PI3K, p-PI3K, mTOR, p-mTOR, ERK1/2, pERK1/2, MEK, pMEK, LaminA, Flag and GAPDH overnight at 4 °C. Details of the antibodies used are shown in Supplementary Table S2. In particular, anti-TACC1 (ab17915, Abcam, Cambridge, UK), was against TACC1 containing mature exon 5 s and the TACC domain.

### Verification of TACC1v25 expression using TCGA RNA-seq data from HNSCC samples

HNSCC samples were extracted from the SpliceSeq database (http://bioinformatics.mdanderson.org/TCGASpliceSeq/), including 387 samples (351 HNSCC and 36 normal samples) after excluding missing values. Data on percent-splice-in (PSI), the ratio of normalized read counts indicating the inclusion of a transcript element over the total normalized reads for that event, were collected from the database. The percentage of samples with a PSI value ≥75% was set as a filter to obtain a more reliable set of AS events. Modes of AS events were previously defined as follows: AA, AD, AP, AT, ES, ME and RI.12. The analysed HNSCC samples originated from TCGA and included 564 samples (520 primary HNSCC and 44 normal samples). Normalized expression levels of TACC1v25 in HNSCC were collected from the TSVdb online tool (http://www.tsvdb.com). Data were processed using GraphPad Prism 8.0.1.

### Scansite analysis

The amino acid sequence data of human TACC1 variant25 (581aa) and full-length TACC1 (851aa) protein were obtained from NCBI database. Scansite (https://scansite4.mit.edu/) was used to predict the phosphorylation sites of TACC1v25 and full-length TACC1 protein.

### IF assay

Cells were plated on coverslips at a density of 3 × 10^4^ cells/ml in 24-well plates. After 24 h, cells were successively treated with PBS, 4% PFA, 0.5% Triton-X, 3% H_2_O_2_ and 3−5% BSA. Cells were incubated with anti-TACC1 antibodies including anti-TACC1 from Abcam (ab17915, Abcam, Cambridge, UK), which was against TACC1 variants containing mature exon 5 s and TACC domain, and anti-TACC1 from Proteintech (Proteintech, China), which was against the SPAZ region of TACC1, and anti-Flag antibody (Proteintech, China). Then, following incubation with Rhodamine (TRITC) goat Anti-Rabbit IgG (H + L; Yeasen, China), the cells were incubated with DAPI for 5 min, and the coverslips were visualized under fluorescence microscopy.

### Cell Counting Kit 8 assay

Cell proliferation was measured using the Cell Counting Kit 8 (CCK8) assay (DOJINDO, Japan) according to the manufacturer’s instructions.

### Mitochondrial membrane potential detection

Mitochondrial membrane potential was assessed using JC-1 (KeyGEN BioTECH, China) according to the manufacturer’s instructions.

### TUNEL assay

The TUNEL assay was performed using the the dT-mediated dUTP Nick-End Labelling kit (TUNEL, KeyGEN BioTECH, China) according to the manufacturer’s instructions.

### TEM

Cells were harvested and fixed in 2.5% glutaraldehyde overnight at 4 °C. After washing with PB three times, cells were fixed in 0.5% osmium tetroxide for 3 h. Samples were embedded in resin and cut into 70 nm sections. Then, the sections were stained with uranyl acetate and lead citrate. Autophagy, morphology and quantity of autophagosomes in the sections were examined by TEM (JEOL JEM1400, Japan). The number of autophagosomes was calculated by randomly selecting five cells in each group.

### Xenograft experiments

Xenograft experiments were performed as previously described [48]. Briefly, 20 specific pathogen-free (SPF) BALB/c-nu male nude mice (14–18 g, 3–5 weeks old; GemPharmatech, China) were randomly divided into Lenti-TACC1v25 group and Lenti-NC group with five mice in each group. Then, 1 × 107 Lenti-TACC1v25 and Lenti-NC Cal27/Fadu cells were resuspended in 200 µl PBS and injected into the right flank of each mouse, respectively. Tumour size was recorded every 3 days. Tumours were extracted and measured 21 days after injection. The experiment was approved by the institutional review board (#SH9H-2020-A430-1).

### Statistical analysis

PSI was calculated to evaluate possible splice events in HNSCC. The difference in TACC1v25 expression between primary HNSCC and normal samples from TCGA was compared using a nonparametric Mann-Whitney test. *P* < 0.05 was considered to indicate a statistically significant difference.

## Supplementary information

Supplementary materials.

Reproducibility Checklist.

## Data Availability

All data generated or analysed during this study are included in this article and its supplementary information files.
