# Supplementary material for: Loss of TACC1 variant25 inducing cell proliferation and suppressing autophagy in head and neck squamous carcinoma
Source: Cell Death Discov. 2021 Dec 11;7:386. doi: 10.1038/s41420-021-00777-6 (PMC8665927; doi:10.1038/s41420-021-00777-6)
Supplement: Supplementary file 1 — Supplementary materials. [file 41420_2021_777_MOESM1_ESM.pdf]

Table S1 Primers detected about TACC1 variants

PCR		
TACC 1 variant s	Forward primers（5' -3' ）	Reverse primers（3' -5' ）
1	CGTCTTCCCGGCTAGTGGAG	TCTGGCACGTCTCCTTCTCT
2	CTTGAGTTTCAGGAGTGGCTG TAA	CTCTGGCTCACCTTTCACCTC
3	GCCGAAGAGCTCGGATTCTG	CTTGTCCTTTGTTCATCTTCAA TCA
4	GCCGAAGAGCTCGGATTCTG	CTTGTCCTTTGTTCATCTTCAA TCA
5&7	GAAGAGTCCCAAGGGTTCCA	TCCTGCCAATCCGAGTGATG
6	CGTCTTCCCGGCTAGTGGAG	TCTGGCACGTCTCCTTCTCT
8	GTCGCGTTTAATAACGACTAC TGA	TCTATGCCTTTCACCTCGGC
9	CGGAGACCGCCTCGGATTC	GCCTGCTTCTGAGGATGAAA
10	AGCAGCAGAGGTCTAGCA	TCTATGCCTTTCACCTCGGC
11	CGGAGACCGCCTCGGATTC	ACGTCTCCTTCTCTATGCCTTT
12&14	GAAGAGTCCCAAGGGTTCCA	TCCTGCCAATCCGAGTGATG
13	ATGCGAAATCAGCGGTAGCA	GTTCTCCAAGCCAACATGCAA
15	CGCCGAAGAGCTCGGATTC	TTGTTCAGTAGTCGTTATTAAA CGC
16	TCGCCGAAGAGAATCACAA	TTGTCCTTTGTTCATCTTCAAT CA
17	GGAGACCGCAATCACAAGA	GCCTGCTTCTGAGGATGAAA
18&29	CTTTTGCCTCCTTAACTGGA	TCTTGACTGAGTGAGAGTCA
19	AGTGACACCAACGACTCAGG	GCTTCTTCACCTTACAGCCAGT TA
20	TCGCCGAAGAGAATCACAA	CCTTTGTTCATCAATCATTTGA GCA
21	AAGAGCTCGGATTCTGAAGG T	CCTTTGTTCATCAATCATTTGA GCA
22	TCGCCGAAGAGAATCACAA	ACGTCTCCTTCTCTATGCCTTT
23	GGAGACCGCAATCACAAGA	ACGTCTCCTTCTCTATGCCTTT
24	GAACCTGAAGTTTTACTGGCT	ATCCGTGGAATCCTTCGTTT

	C	
25	GGTGAAGAAGGTCGCGTGAT	TTCCGAGGACTGCCGAGATA
26	GCCGAGGTGAAAGGCATAGA	CCTTTGTTCATCAATCATTTGAGCA
27	CAGAAGCAGGTATGGAAGCA	AAGCAAAGCATAGGTCAGCA
28	GTCACCGGTTCCCTCCATTT	TCAGTAGTCCTGAAACTCAAGG
30	TTGAGTTTCAGGAGTGGCTGT	TCTATGCCTTTCACCTCGGC
31	GAGGCTCCCACTCTCAGACC	TGATTCGCTGAGGCAGATCC
GAPDH	GGACCTGACCTGCCGTCTAG	GTAGCCCAGGATGCCCTTGA
RT-qPCR		
Genes	Forward primers（5' -3' ）	Reverse primers（3' -5' ）
C-FOS	CTTATCTGTGCGTGAAACAC	ACTGGGAACAATACACACTC
c-Jun	AGAAAGTCATGAACCACGTT	CTCAAGTCTGTCTCTCTGTG
DUSP5	ACAGCCCTGCTGAATGTCTC	GGAGCTAATGTCAGCCGTGT
DUSP6	ACTGGAACGAGAATACGGGC	GATTGGTCTCGCAATGCAGG
GAPDH	GGACCTGACCTGCCGTCTAG	GTAGCCCAGGATGCCCTTGA

Table S2 Antibodies used

Antibody	Catalog NO.	Brand	City	Country
p-AKT (ser473)	#4691	CST	Massachusetts	America
AKT	#4058	CST	Massachusetts	America
p-mTOR (ser2448)	#5536	CST	Massachusetts	America
mTOR	#2983	CST	Massachusetts	America
PI3K	#4257	CST	Massachusetts	America
p-PI3K	#4228	CST	Massachusetts	America
Beclin-1	#3495	CST	Massachusetts	America
LC3B	#43566	CST	Massachusetts	America
BCL-2	#15071	CST	Massachusetts	America
ERK1/2	#4695	CST	Massachusetts	America
p-ERK1/2	#4377	CST	Massachusetts	America
MEK	#8727	CST	Massachusetts	America
p-MEK	#9154	CST	Massachusetts	America
LaminA	Ab26300	Abcam	Cambridge	Britain
Flag	20543-1-AP	Proteintech	Chicago	America
GAPDH	AP0066	Bioworld	Minnesota	America
Anti-TACC1 antibody	ab17915	Abcam	Cambridge	Britain
TACC1 Polyclonal antibody	13862-1-AP	Proteintech	Chicago	America
HRP-conjugated Affinipure Goat Anti-Mouse IgG(H+L)	#7076	CST	Massachusetts	America
HRP-conjugated Affinipure Goat Anti-Rabbit IgG(H+L)	33101ES60	YEASEN	Shanghai	China

Table S3 AS events of TACC1 from TCGA-HNSC

Symbol_ID	AS_ID	Splice_Type	Exon	PSI_average
TACC1-AP-83434	83434	AP	1	0.661
TACC1-AP-83435	83435	AP	1a	0.160
TACC1-AP-83436	83436	AP	X5	0.061
TACC1-AP-83437	83437	AP	1b	0.118
TACC1-AT-83438	83438	AT	13	0.996
TACC1-AA-83440	83440	AA	10	0.972
TACC1-AD-83441	83441	AD	5	0.674
TACC1-AA-83443	83443	AA	4	0.999
TACC1-ES-83445	83445	ES	4	0.999
TACC1-ES-83447	83447	ES	4a	0.053
TACC1-ES-83449	83449	ES	2-3	0.943

Figure S1 Raw Western Blot of Figure 2A

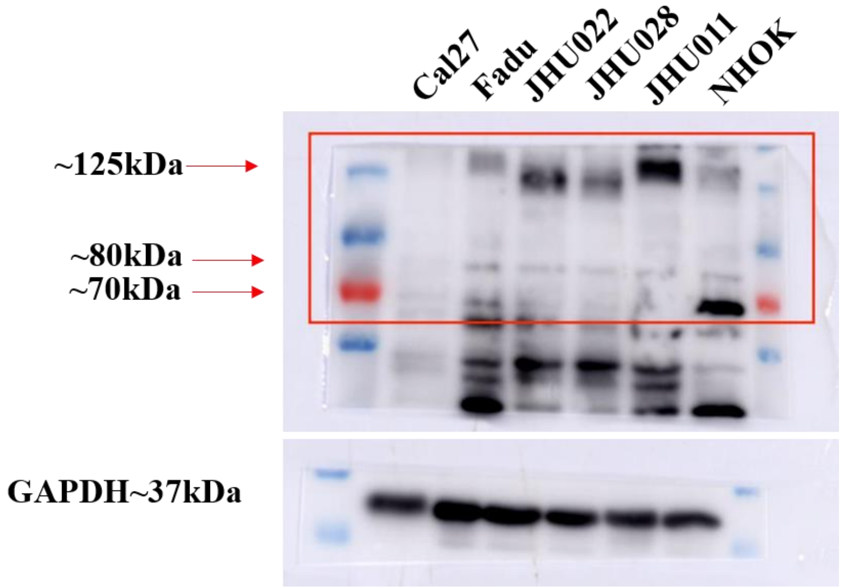

Western blot analysis. Because the anti-TACC1 domain antibody identifiesall TACC1 variants proteins, stronger bands at ~70 kDa and ~80 kDa in NHOK were TACC1v25 and /or 27 combined with RT-PCR.
